# Identification of immunologic subtype and prognosis of GBM based on TNFSF14 and immune checkpoint gene expression profiling

**DOI:** 10.18632/aging.103065

**Published:** 2020-04-20

**Authors:** Shengrong Long, Mingdong Li, Jia Liu, Yi Yang, Guangyu Li

**Affiliations:** 1 Department of Neurosurgery, First Affiliated Hospital of China Medical University, Shenyang, China

**Keywords:** glioblastoma, prognosis, IGCs, TNFSF14, immunologic subtype

## Abstract

Immune-checkpoint therapy has failed to show significant benefit in glioblastoma (GBM) patients. Immunologic subtypes of GBM are necessary to identify patients who might benefit from immune-checkpoint therapy. This study reviewed 152 GBM samples from The Cancer Genome Atlas (TCGA) and 214 GBM samples from Chinese Glioma Genome Atlas (CGGA). Correlation analysis showed that immune checkpoint genes (ICGs) were mainly positively correlated. The prognostic analysis of the overall survival showed that there was a significant correlation between the overall survival (OS) and the prognosis of ICGs, in which the TNFSF14 gene was a significant adverse prognostic factor. Combined with TMB and neoantigens, we found that TNFSF9 and CD27 were significantly negatively correlated with TMB and neoantigens. The association between adaptive immune pathway genes and ICG expression showed that they were positively correlated with ICGs, indicating that adaptive immune pathway genes have a certain regulatory effect on the expression of ICGs. The analysis of clinical features of the samples showed that the higher the expression of ICGs, the more likely to be correlated with mutant isocitrate dehydrogenase (IDH), while the lower the expression level of IDH, the more likely to be significantly correlated with the primary GBM. Survival analysis showed that low expression of PD-L1, IDO1, or CTLA4 with TNFSF14 in the low expression group had the best prognosis, while high expression of IDO1 or CD274 with TNFSF14 in the high expression group and low expression of CTLA4 with TNFSF14 in the high expression group had the worst prognosis. We conclude that TNFSF14 is a biomarker to identify immunologic subtype and prognosis with other ICGs in GBM and may serve as a potential therapeutic target.

## INTRODUCTION

Gliomas are derived from glial cells and are the most prevalent type of primary tumor in the human brain. The World Health Organization (WHO) proposed a four-grade classification system for gliomas, low-grade gliomas (LGG, grades I–II), and high-grade gliomas (HGG, grades III–IV). WHO grade IV glioma, also known as glioblastoma or glioblastoma multiforme (GBM), is characterized by ischemic necrosis, invasiveness, and microvascular hyperplasia [1–3]. The incidence of GBM is 0.59–3.69/100,000 worldwide, and the median age of onset is 63.0 years. The age-adjusted incidence was 3.97 per 100,000 males and 2.53 per 100,000 females [4–6]. Standard therapies according to National Comprehensive Cancer Network (NCCN) guidelines include tumor resection, radiotherapy with concomitant temozolomide (TMZ), and adjuvant TMZ chemotherapy, with the combination of radiotherapy and other therapies. GBM patients have a very poor prognosis after routine surgery, radiotherapy, and chemotherapy. The five-year overall survival (OS) rate was 9.8%, while that of radiotherapy alone was 1.9%. Despite standard treatment, the median survival time was only 12–15 months after diagnosis [1, 2]. The insufficiency of conventional oncology treatment has prompted researchers to look for more targeted treatment strategies. In recent years, immunotherapy, including immune checkpoint blocking, chimeric antigen receptor T-cell therapy (CAR-T) and dendritic cell (DC) therapy, have introduced hope for glioma patients [7].

Immune checkpoints are co-stimulators or co-suppressors required to produce an immune response [8]. Blocking co-inhibitory checkpoints, such as cytotoxic T-lymphocyte-associated protein 4 (CTLA-4) and programmed cell death protein 1 (PD-1)/PD-L1, were breakthroughs in the treatment of a variety of malignant tumors [9]. For example, PD-1/PD-L1 checkpoint blocking greatly changed the treatment of non-small cell lung cancer (NSCLC), kidney cancer, chronic Hodgkin's lymphoma, gastric cancer, head and neck squamous cell carcinoma, hepatocellular carcinoma, and melanoma [10–14]. Several studies have shown that PD-L1 is highly expressed in GBM, and checkpoint blocking immunotherapy has shown positive results in a GBM mouse model, suggesting that immune checkpoints can be used in the treatment of GBM [15–17].

Despite the fact that several new immunotherapy approaches for glioma have emerged, the effect of these new treatments remains unsatisfactory [18, 19]. Therefore, there is an urgent need to understand the immune status of gliomas to identify more effective treatments for this refractory disease. In glioma, various immune cells and stroma constitute non-tumor components of the tumor parenchyma, including T-cells, tumor-associated macrophages (TAMs) and natural killer (NK) cells. Through the secretion of cytokines or ligand-receptor interaction, they form microenvironments conducive to malignant progression of glioma [20]. The efficacy of glioma immunotherapy for PD-1 has been confirmed in only some glioma patients, and it is often accompanied by inflammation and immune-related side effects [18].

There is an urgent need to identify effective checkpoints other than PD-1/PD-L1 that participate in GBM progression. Therefore, in this study, we analyzed expression patterns of 47 known immune checkpoint genes (ICGs) and their association with prognosis. Then, the relationship between immunotherapy biomarkers and ICGs, such as mismatch repair defect (MMRd) and tumor mutation burden (TMB), was studied using integrated somatic mutation data to determine the relationship between the expression of ICGs (PD1/PD-L1, CTLA4) and other biomarkers that are widely used as immunotherapy. Finally, we studied the association between ICGs and immune activation-related signature genes to determine the relationship between immune activation and immunosuppression in the tumor microenvironment.

## RESULTS

We obtained 152 samples from TCGA and 214 samples from CGCA after preprocessing. The clinical information from the two datasets after preprocessing are displayed in Table 1.

**Table 1:** Clinical information of datasets of TCGA and CGGA after preprocessing.

r**TCGA**		**CGGA**	
SEX		SEX	
Male	98	Male	127
Female	54	Female	87
PFS		OS	
30 ~925	138	30 ~925	180
925 ~1820	12	925~1820	25
1820 ~2681	2	1820~2863	9
Event		Event	
Dead	122	Dead	166
Alive	30	Alive	48
New Event		PRS type	
0	59	Primary	132
1	93	Recurrent	82
		IDH mutation status	
		Mutant	39
		Wildtype	167

### Association between ICGs and prognosis of GBM in TCGA

**Figure 1:**
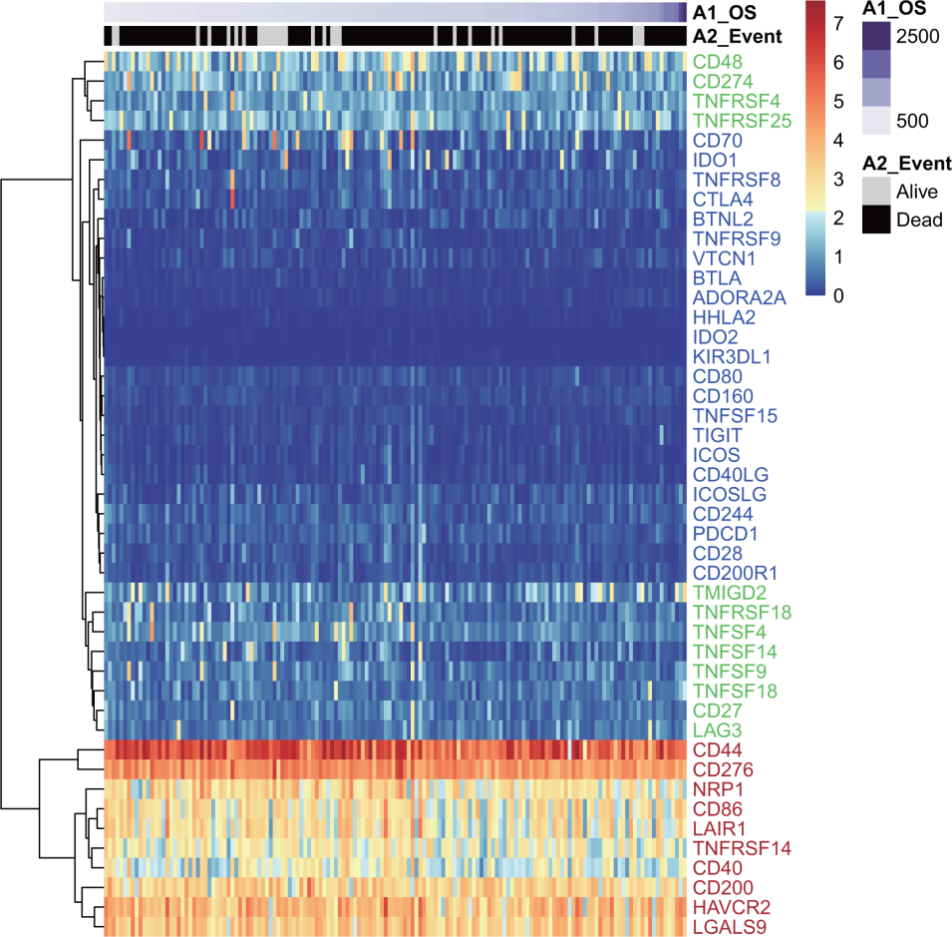
**The heatmap of ICG expression in the TCGA-GBM dataset.** Red indicates the high expression group; green indicates the moderate expression group; blue indicates the low expression group.

Forty-five of 47 ICGs were expressed in the TCGA GBM dataset (Figure 1). Depending on expression level, these 45 ICGs could be divided into three expression groups: high expression group (red), moderate expression group (green), and low expression group (blue). The high expression group was represented by CD44 and CD276, and the expression level was generally high in all samples, while the moderate expression group was represented by CD48 and CD274 (PD-L1). The expression levels were significantly different among samples in the moderate expression group. The low expression group was represented by IDO2 and HHLA2, and the expression levels were lower in most samples.

### Prognostic analysis of ICGs

**Figure 2:**
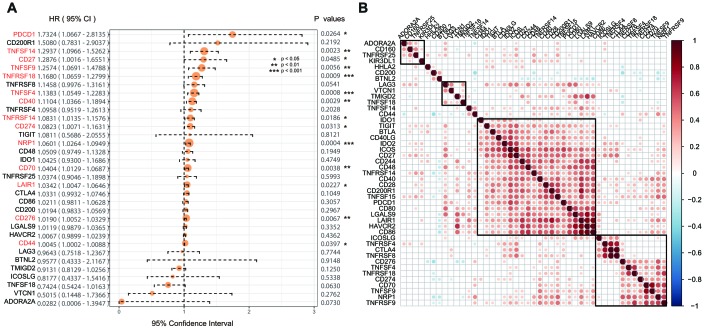
(**A**) The prognostic effect analyses of ICGs expression commonly used prognostic factors using univariate Cox regression model in the TCGA-GBM dataset. (**B**) Correlation of ICGs expression level in TCGA. Note: Only the correlation test significant gene pairs were shown. The blank means that the correlation test is not significant.

Univariate Cox regression analysis was used to calculate the associations between these 45 ICGs and GBM prognosis. A total of 14 ICGs significantly correlated with prognosis (Figure 2A, log rank p < 0. 05). Correlation analysis of ICG expression levels showed that there were mainly positive correlations, and there was a substantial aggregation effect (Figure 2B), suggesting synergistic expression associations among these ICGs.

### Association between ICGs and prognosis of GBM in CGGA

**Figure 3:**
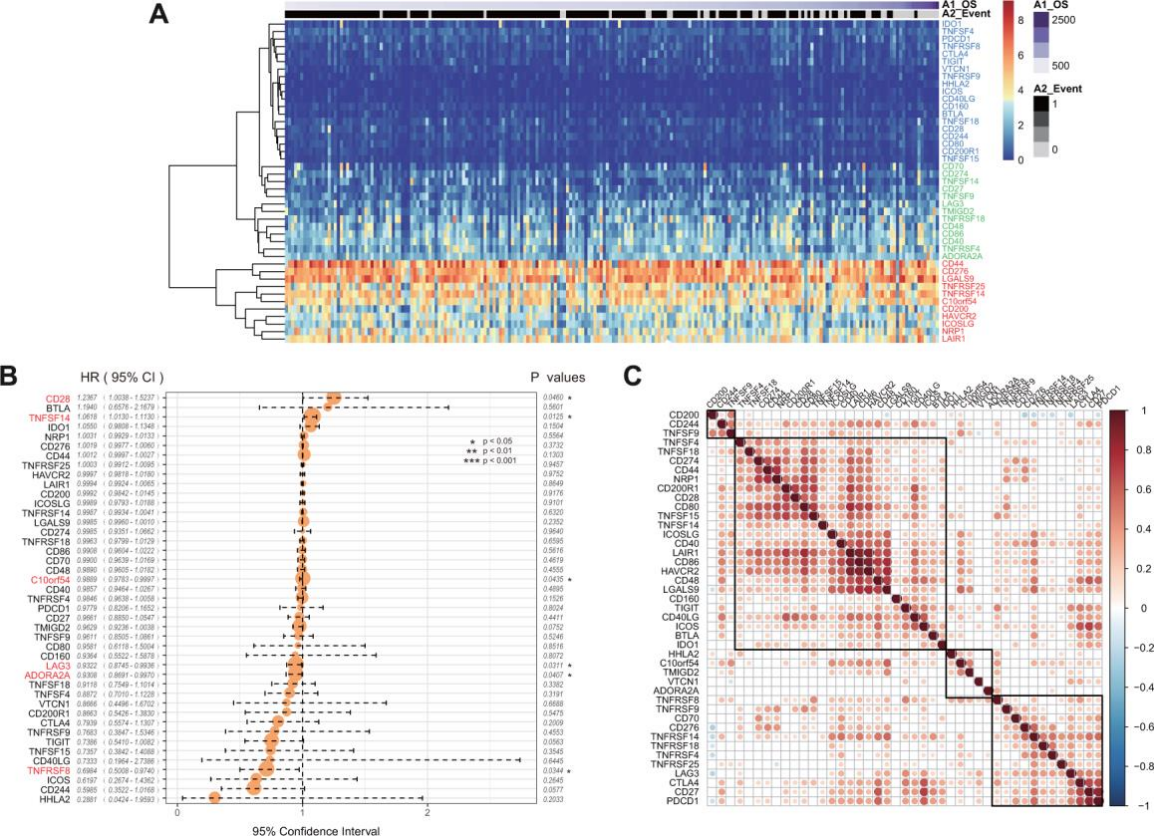
(**A**) The heatmap of ICGs expression in CGGA-GBM dataset. Red indicates the high expression group; green indicates the moderate expression group; blue indicates the low expression group. (**B**) The prognostic effect analyses of ICGs expression commonly used prognostic factors using univariate Cox regression model in CGGA-GBM dataset. (**C**) Correlation of ICGs expression level in CGGA. Note: Only the correlation test significant gene pairs were shown. The blank means that the correlation test is not significant.

Forty-three of 47 ICGs were expressed in the CGGA GBM dataset. These were divided into three groups: high expression group, moderate expression group, and low expression group (Figure 3A). We found that this distribution group was highly consistent with those of the three expression groups of TCGA. For example, CD44, CD276, and NRP were divided into high expression groups in the TCGA and CGGA datasets. However, CD48, CD274, and TNFRSF4 showed moderate expression levels in the two datasets, while IDO1, TNFRSF8 and CTLA4 showed low expression levels in the two datasets.

Univariate Cox regression analysis showed that there was a significant association between six ICGs and the prognosis of OS (Figure 3B, log rank p < 0. 05). The TNFSF14 gene was significantly associated with poor prognosis in both TCGA and CGGA datasets (HR > 1, log rank p < 0.005). In the CGGA dataset, expression levels of ICGs was also positively correlated, and there was a substantial aggregation effect (Figure 3C) that was consistent with the results of TCGA.

### Association between ICGs and TMB

**Figure 4:**
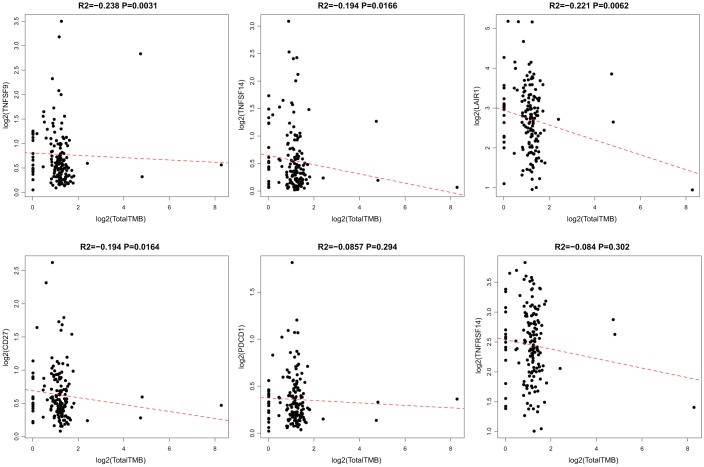
**Pairwise Pearson correlation between immune markers and TMB in TCGA.** Scatter plots of pairwise expression between ICGs. The Pearson correlation coefficient (R2) and corresponding P value are shown at the top of each plot.

According to somatic mutation data from TCGA, we calculated the TMB of GBM. We removed the intron interval and annotated silent mutation when calculating TMB. First, 14 ICG expressions that were significantly related to OS were chosen. The correlation between TMB and these 14 ICGs was evaluated using the Spearman approach (the distribution of TMB did not satisfy normal distribution: Shapiro test p < 10–5). ICG in TMB data are shown in Supplementary Table 4. We found that there was a significant negative correlation between TMB and the expression of TNFSF9, TNFSF14, LAIR1, and CD27 (Figure 4, R2 < 0 and p < 0.05). Considering that TNFSF9 and TNFSF14 were extremely significant poor prognostic factors, we speculated that high expression of TNFSF9 and TNFSF14 would correspond to low TMB, while low TMB would not be suitable for immunotherapy. Tumor mutational burden predicts survival after immunotherapy across several cancer types.

### Association between ICGs and neoantigens

**Figure 5:**
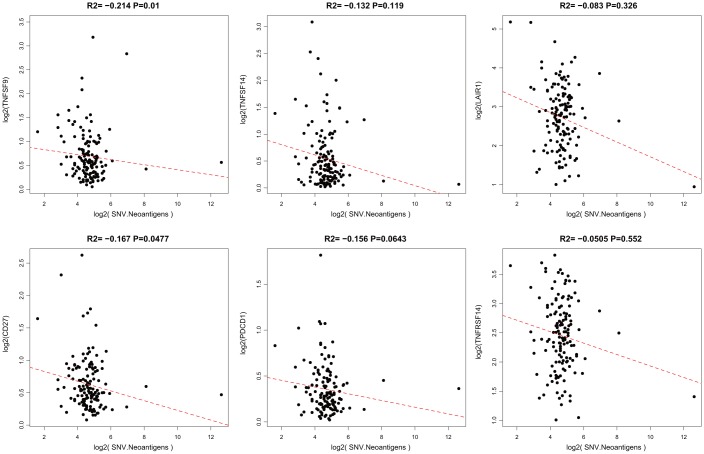
**Pairwise Pearson correlation between immune markers and neoantigens in TCGA.** The Pearson correlation coefficient (R2) and corresponding P value are shown at the top of each plot.

When a tumor somatic mutation appears on a gene that encodes a protein, it produces an erroneous protein (neoantigen). These neoantigens produced by mutations can be presented by the major histocompatibility class I complex (MHC I). These new proteins induce anti-tumor adaptive immune response by binding to T-cell receptors (TCRs). Based on the somatic mutation data in TCGA-GBM, we further analyzed the association between the expression of neoantigens and ICGs (Supplementary Table 5). We found that there was a significant negative correlation between TNFSF9, CD27, and neoantigens (Figure 5, R2 < 0, p < 0.05), consistent with the significant negative correlation between TMB and ICG mentioned above.

### Association between ICGs and adaptive immune-resistance pathway markers

**Figure 6:**
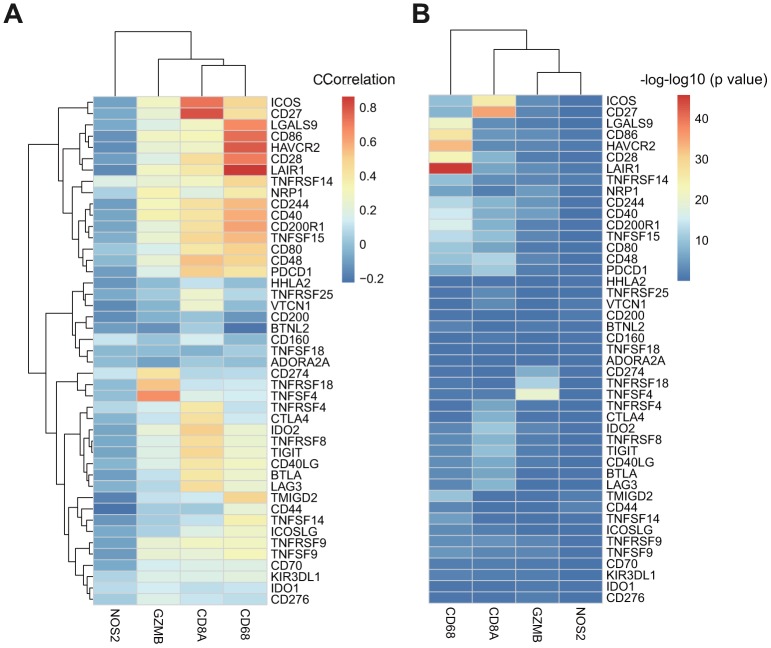
(**A**) The heatmap of correlation coefficient between ICGs and adaptive immune resistance pathway genes in TCGA-GBM. (**B**) The P-value of correlation coefficient Test between ICGs and adaptive immune resistance pathway genes in TCGA-GBM. P-value has been performed as a-log10 conversion.

CD8+ T-cells produce interferon (IFN)-γ that leads to u-regulation of adaptive immune resistance pathway gene expression, including PD-1/PD-L1, IDO1, and others. Therefore, we analyzed associations between CD8A, GZMB, CD68, NOS2, and ICGs (Supplementary Table 6). We found that expression levels of three of four genes in the adaptive immune resistance pathway (except NOS2) highly correlated with the expression of ICGs (Figure 6A), and most correlated positively. The significant test of correlation coefficient showed that most of the correlations between these genes were extremely significant (p < 10–5, Figure 6B).

We also analyzed the expression relationships between adaptive immune-resistance pathway genes and ICGs (Supplementary Table 7) using CGGA data. We found that these two gene groups were positively correlated. CD68 and CD8A showed significant positive correlations with ICOS, CD27, LAIR1, and CD86 (Supplementary Figure 1), consistent with the results of the TCGA dataset.

### Association between ICGs and clinical features

**Figure 7:**
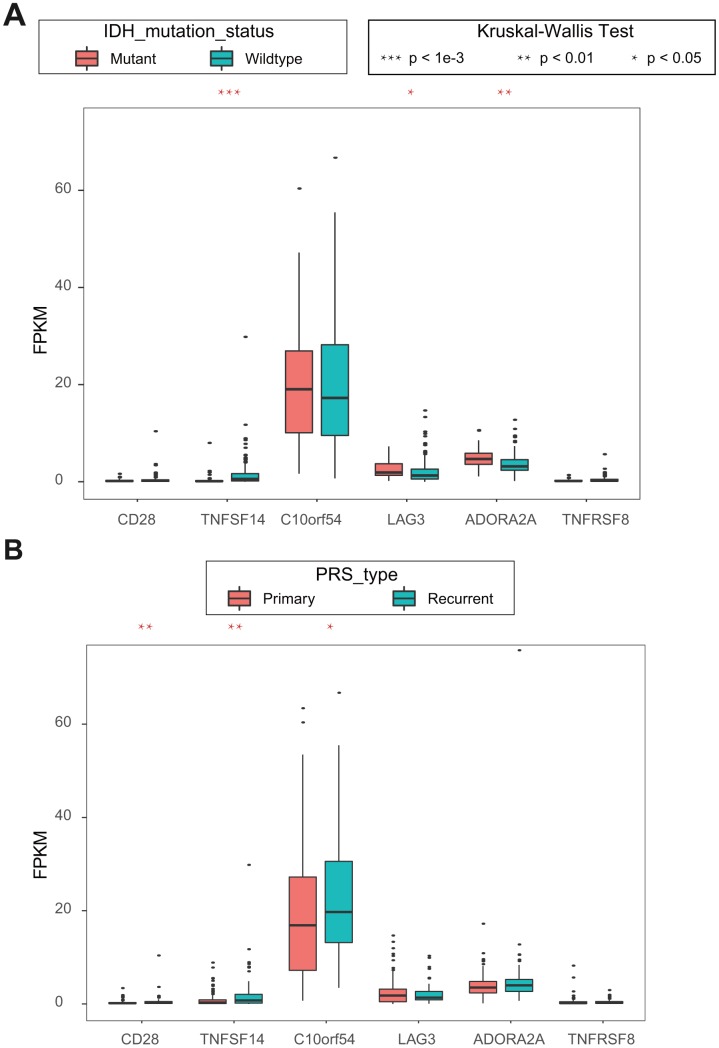
(**A**) Kruskal-Wallis test of expression (FPKM) of 6 ICGs on IDH mutation status; (**B**) Kruskal-Wallis test of expression (FPKM) of 6 ICGs on PRS type**.**

Because there is little clinical information in TCGA, we used univariate Cox regression analysis in CGGA to identify six ICGs with OS prognosis. Combined with the clinical information in CGGA, we analyzed the association between these six ICGs and clinical features, isocitrate dehydrogenase (IDH) mutation status and PRS type. Higher expression levels of ICG significantly correlated mutant IDHs, including TNFSF14, LAG3, and ADORA2A (Figure 7A, 7B). Lower expression levels of IDH significantly correlated with primary tumors, including CD28, TNFSF14, and C10orf54. There was a significant correlation between TNFSF14 and IDH gene mutation and tumor PRS type status.

### Association between prognosis and GBM subtypes defined by IGCs

PD1 (PDCD1), PD-L1 (CD274), and IDO1 are important genes for immunological checkpoints for immunotherapy. Nevertheless, we found that not all of them had a significant prognostic relationship with OS as determined by univariate Cox regression. Based on our analysis, we found that TNFSF14 was not only significantly correlated with the prognosis of TCGA and CGGA samples, but also with TMB. In addition, TNFSF14 was significantly correlated with the important mutant gene IDH in GBM, suggesting that TNFSF14 may cause imbalance of gene expression in the adaptive immune resistance pathway. Therefore, we analyzed the relationship between PD-L1 (CD274), IDO1 combined with TNFSF14, and prognosis.

**Figure 8:**
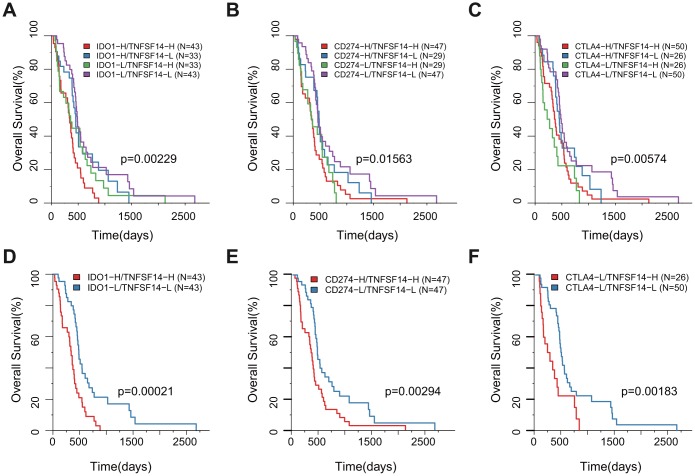
**Survival KM plot by a combined analysis of ICGs and TNFSF14 expression in TCGA.** (**A**) Survival based on high/low IDO-1 expression and TNFSF14 expression. (**B**) Survival based on high/low CD274 expression and TNFSF14 expression. (**C**) Survival based on high/low CTLA-4 expression and TNFSF14 expression. (**D**–**F**): The best and worst prognosis of the above expression combinations.

According to the H/L expression groups of IDO1, CD274, and CTLA4 combined with TNFSF14, we divided the GBM samples into four combinations based on the median of gene expression for all three pairs of genes. The survival analysis of the three pairs showed that there was a significant difference in the OS among the four high- and low-expression combinations. The group with low expression of TNFSF14 with low expression of IDO1, CD274, or CTLA4 had the best prognosis, while the groups with high expression of TNFSF14 with high expression of IDO1 and CD274 and high expression of TNFSF14 with low expression of CTLA4 had the worst prognosis (Figure 8A–8C). Taking the two groups of samples with the best prognosis and the worst prognosis to analyze the prognosis of OS, we found that there was a significant difference between the two groups (Figure 8D–8F).

Based on the CGGA dataset, we also observed a partial significant association between differential expression of IDO1, CD274, or CTLA4 with H/L expression of TNFSF14 and prognosis (Supplementary Figure 2).

### IHC

**Figure 9:**
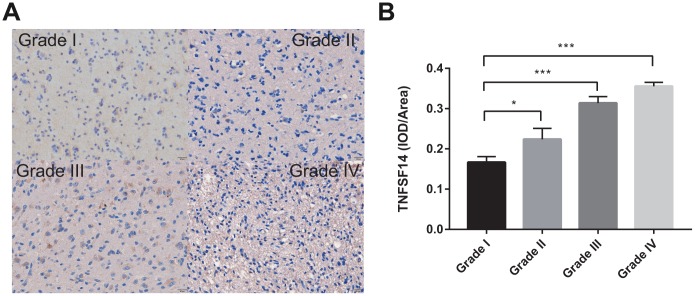
**Levels of TNFSF14 expression relative to common pathology in glioma tissue.** (**A**) Levels of expression and representative photographs of immunohistochemical staining of TNFSF14 in different grades of gliomas. (**B**) Quantitative bar graphs of immunohistochemical staining in each grade.

To further investigate the clinical significance of TNFSF14 in glioma, we analyzed the expression of TNFSF14 according to grade of glioma. We found that TNFSF14 expression level increased with increasing glioma grade (Figure 9). The results suggest that TNFSF14 is associated with higher tumor malignancy.

## DISCUSSION

GBM is a malignant tumor characterized by a variety of immune cell infiltrations and accompanied by the formation of complex immune responses. The use of immune checkpoint inhibitors to benefit patients with GBM may be a novel method because these patients have a poor prognosis. The conventional belief that the blood–brain barrier offers immune privilege no longer seems accurate, suggesting that immunotherapy could have benefits in CNS cancer [21]. Immunotherapy includes vaccine therapy, checkpoint inhibitors, chimeric antigen tcr, and viral immunotherapy, which can improve prognosis and OS for GBM patients [22]. The immune checkpoint inhibitors have improved survival in treatment-resistant solid tumors, including melanoma, NSCLC, and renal cell carcinoma (RCC) [23]. Immunomodulation between immune cells or between tumor cells and immune cells promotes tumor progression [24]. Preclinical studies have shown that immunotherapy-based methods have been successful in animal models. Several phase I and phase II clinical trials have shown that immunotherapy is safe and in some cases can improve progression-free survival (PFS) and OS [25–29]. Immune checkpoints are key molecules for immunoregulation. Checkpoint inhibitor therapy showed great potential for immunotherapy of gliomas [30]. Although novel immune-checkpoint inhibitors targeting PD-1, PD-L1, or CTLA-4 have activity in many different cancers, they have thus far failed to show meaningful clinical benefit in unselected patients with GBM. The objective response rate to immune-checkpoint therapy in unselected patients with GBM has been approximately 5% in prior clinical trials. Based on its poor response rate to current systemic immunotherapies, GBM is largely considered to be a poorly immunogenic cancer type. To optimally identify patients who may have intact antitumor immune responses and/or benefit from immunotherapeutic strategies, an improved immunologic characterization of GBMs is needed. Preclinical studies using mouse models with orthotopically transplanted gliomas have shown great benefits with checkpoint inhibitors alone or with other immunotherapeutic strategies [31–34].

Immune checkpoint inhibitors, therefore, are among the promising methods to activate anti-tumor immune response, showing remarkable success in a variety of cancer treatments [35, 36].

In this study, we analyzed expression of 47 ICGs and the relationship with prognosis. The OS rate significantly correlated with prognosis of ICGs; TNFSF14 was a significant adverse prognostic factor in both the TCGA and CGGA datasets (HR > 1, log rank p < 0.05).

Co-stimulation plays an important role in the activation of T-cell-mediated immunity. Several studies have demonstrated that ligand-receptor pairs of the tumor necrosis factor (TNF)/TNF receptor family are important regulators of this process [37]. At least five ligand-receptor pairs of the TNF-TNF receptor family have been shown to affect T-cell activation at early or late stages after antigen encounter: OX40-OX40L, CD27-CD70, herpesvirus entry medium (HVEM)-LIGHT, and CD30-CD30L. Among these costimulatory molecules, LIGHT/TNFSF14 plays a key role in regulating T-cell function and promoting T-cell mediated anti-tumor immunity [38–40].

LIGHT is mainly produced by T-cells and monocytes, and its effect is mediated by three receptors: lymphotoxin β-receptor (LT β-R), HVEM, and decoy receptor 3 (DcR3) that inhibit its function [38, 41]. Previous studies have shown that LIGHT-deficient macrophages display reduced production of cytokines, while constitutive overexpression of LIGHT promotes T lymphocyte activation and maturation and leads to severe inflammation and tissue destruction, resulting in severe inflammation and tissue damage [42, 43].

In this study, we observed a significant negative correlation between the expression of TNFSF14 and TMB (Figure 4, R2 < 0, p < 0.05). Because TNFSF14 is a significant poor prognostic factor for OS, we speculate that the high expression of TNFSF14 corresponds to low TMB; low TMB is not suitable for immune checkpoint inhibitor therapy. Nevertheless, the correlation analysis between adaptive immune pathway genes (CD8A, CD68, GZMB, NOS2) and ICG expression showed that there were significant positive correlations with most ICGs, suggesting that adaptive immune pathway genes have a regulatory effect on the expression of ICGs. Furthermore, they are significantly correlated with IDH, an important mutation gene in GBM, suggesting that TNFSF14 may cause imbalance of gene expression in the adaptive immune resistance pathway. Therefore, we speculated that there may be a correlation between TNFSF14 and immune checkpoint. Under normal circumstances, the immune system reacts to foreign antigenic substances gathered in the lymph nodes or spleen, resulting in an increase in the number of antigen-specific cytotoxic T cells. The combination of PD-1 and PD-L1 can transmit inhibitory signals and inhibit the proliferation of T cells. Increased human T cell infiltration can upregulate the expression of IDO1 in GBM, while the high expression of IDO1 usually indicates a poor prognosis of GBM. There is a certain correlation between biomarkers currently used in immunotherapy, which may be attributed to T cell infiltration to some extent [44]. We analyzed the relationship between the combination of PD-L1 (CD274), IDO1 with TNFSF14, and prognosis of GBM in both TCGA and CGGA. Integrating the expression levels of PD-L1, IDO1, CTLA4, and TNFSF14 to group GBM samples, the samples can be divided into four combinations. Survival analysis showed that the prognosis was the best in the combinations of low expression of PD-L1, IDO,1 and CTLA4 with low expression of TNFSF14, while the combinations of high expression of IDO1 and CD274 with high expression of TNFSF14 and the combinations of low expression of CTLA4 with high expression of TNFSF14 had the worst prognosis. Immune checkpoints have been shown to play an immunosuppressive role in cancer. These checkpoints, including CTLA-4, PD-1/PDL1/2, TIM-3, ICOS, TIGIT, and CD96, are being studied, and new inhibition pathways are being developed. By analyzing the relationship between the combination of PD-L1 (CD274), CTLA-4, IDO1 with TNFSF14, and prognosis, we found that TNFSF14 plays a significant role in determining the survival prognosis of patients with GBM for these immune checkpoints that are currently widely used in immunotherapy. This result was consistent with that of Wu *et al*. They found that ICOs, TNFSF14, and ULBP1 were the important immune checkpoints in GBM [45].

In summary, we analyzed the expression of 47 ICGs and the relationship between their expression and prognosis in GBM. Then, the relationship between immunotherapy biomarkers and ICGs, including MMRd and TMB, was studied using integrated somatic mutation data. We found that TNFSF14 can cause imbalance of adaptive immune resistance pathway gene expression. In combination with ICG analysis of biomarkers, we identified the importance of TNFSF14 in the survival and prognosis of GBM in immunity. This finding may suggest new immunotherapy strategies for treatment of GBM and may provide a new target for diagnosis and treatment of GBM in the future.

Our methodology relies on the availability of multidimensional data, but there are only IHC results and clinical datasets for GBM. Future experiments will involve TNFSF14 in *vivo* and in *vitro*. These will further improve the predictive power of our approach.

## MATERIALS AND METHODS

### Sources of ICGs

A total of 47 immune checkpoint genes are shown in Supplementary Table 1.

### The cancer genome atlas (TCGA) and chinese glioma genome atlas (CGGA) data

We used TCGA GDC API to download the latest clinical follow-up information and mRNA-Seq data from the TCGA-GBM dataset. We obtained a total of 160 samples. The mRNA-seq data in FPKM format were downloaded from the CGGA, including 693 glioma samples accompanied by clinical characteristics. We extracted 249/693 samples with grade IV as GBM samples. The relevant data are displayed in Supplementary Tables 2, 3.

### Preprocessing of raw data

### TCGA data preprocessing

The following steps were performed on 160 GBM samples:

1. Removal of samples without clinical information or OS < 30 days.
2. Removal of normal tissue sample data.
3. Removal of genes with fragments per kilobase per million (FPKM) = 0 in more than half of the samples.

### CGGA data preprocessing

The RNA-seq data of 249 samples were preprocessed in the following steps:

1. Removal of normal tissue samples and retention of only primary tumor data.
2. Conversion of OS data from years or months to days.
3. Using the R/Bioconductor packages, chip probes were mapped to human gene SYMBOL.
4. Retention only of expression profiles of immune-related genes.

### Immunohistochemistry

Glioma tissues were collected from the First Hospital of China Medical University. This study was approved by the ethics committee of the First Hospital of China Medical University (IRB No: 2017-98-2). All patients signed the informed consent. The expression of TNFSF14 in paraffin-embedded tissues was detected by immunohistochemistry (IHC). Incubation of primary antibody (bs-2462R, IHC-P=1:100-500) was conducted overnight at 4°C. Incubation of secondary antibody was applied for 2 hours at room temperature. Then, the Elite Vector staining ABC system was used for immune detection. 3,3'-Diaminobenzidine (DAB) was used as the substrate for color visualization. Images were obtained using a Nikon TE-2000 Brightfield microscope. Integrated optical density (IOD) to area ratio was calculated for each marker to assess the staining intensity.

### Bioinformatic and statistical analysis

Data analysis were performed using R software (version 3.6.0) with customary routines. The differentially expressed ICGs between the high, moderate, and low groups in TCGA and CGGA were identified using limma R package. Heatmaps and scatter plots were created using the gplots package in the R package. Univariate Cox regression analysis was used to identify prognostic ICGs. Pearson correlation coefficients were used to calculate correlations. Kruskal-Wallis analysis was performed between mutant and wild-type in IDH mutation status, prime and recurrent in PRS type with IGCs. Kaplan–Meier (KM) survival plots were generated using the survfit function from the R package, and P-values from log-rank tests were reported.

### Availability of data and materials

All data generated or analyzed during this study are included in this published article.

## Supplementary Material

Supplementary Figures

Supplementary Table 1

Supplementary Table 2

Supplementary Table 3

Supplementary Table 4

Supplementary Table 5

Supplementary Table 6

Supplementary Table 7
