# Supplementary material for: Identification of immunologic subtype and prognosis of GBM based on TNFSF14 and immune checkpoint gene expression profiling
Source: Aging (Albany NY). 2020 Apr 20;12(8):7112–28. doi: 10.18632/aging.103065 (PMC7202515; doi:10.18632/aging.103065)
Supplement: Supplementary Figures [file aging-12-103065-s006..pdf]

SUPPLEMENTARY MATERIALS

Supplementary Figures

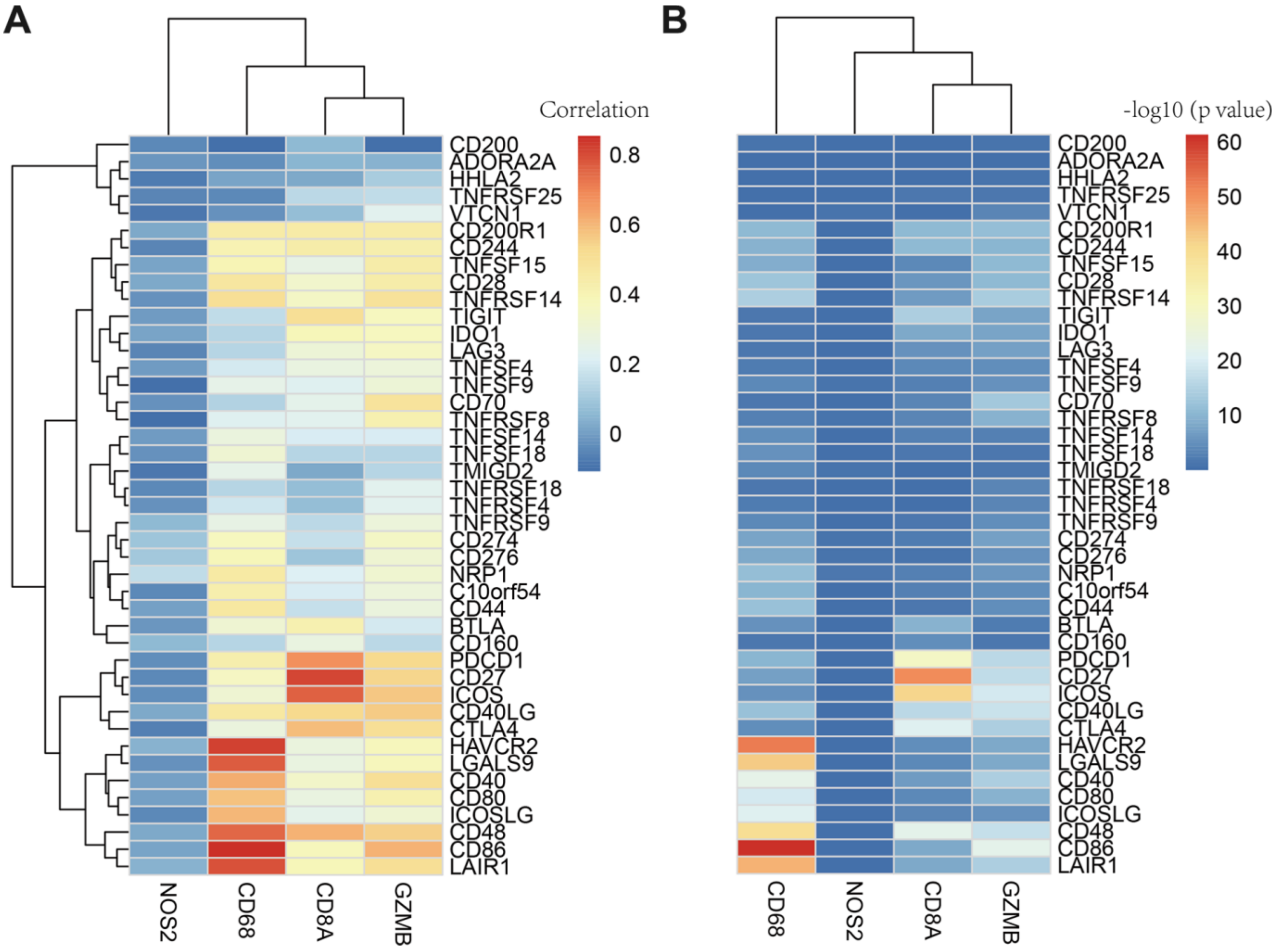

Supplementary Figure 1. (**A**) The heatmap of correlation coefficient between ICGs and adaptive immune resistance pathway genes in CGGA. (**B**) The P-value of correlation coefficient Test between ICGs and adaptive immune resistance pathway genes in CGGA. P-value has been performed as a-log10 conversion.

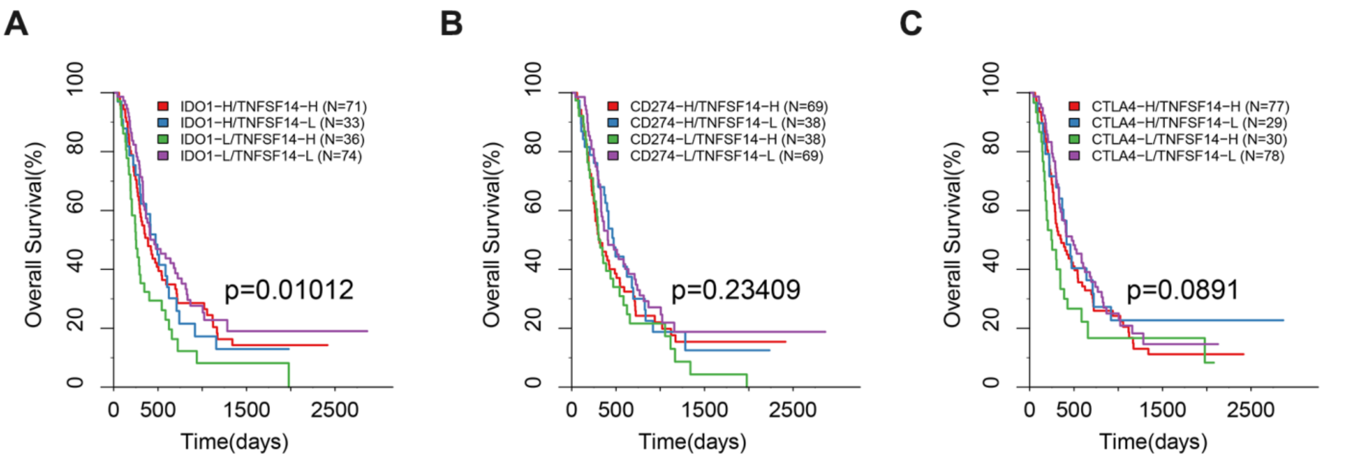

Supplementary Figure 2. Survival KM plot by a combined analysis of ICGs and TNFSF14 expression in CGGA. (**A**) Survival based on high/low IDO-1 expression and TNFSF14 expression. (**B**) Survival based on high/low CD274 expression and TNFSF14 expression. (**C**) Survival based on high/low CTLA-4 expression and TNFSF14 expression.
