# Supplementary material for: Identification of immunologic subtype and prognosis of GBM based on TNFSF14 and immune checkpoint gene expression profiling
Source: Aging (Albany NY). 2020 Apr 20;12(8):7112–28. doi: 10.18632/aging.103065 (PMC7202515; doi:10.18632/aging.103065)
Supplement: Supplementary Table 1 [file aging-12-103065-s003..pdf]

ADORA2A
BTLA
BTNL2
C10orf54
CD160
CD200
CD200R1
CD244
CD27
CD274
CD276
CD28
CD40
CD40LG
CD44
CD48
CD70
CD80
CD86
CTLA4
HAVCR2
HHLA2
ICOS
ICOSLG
IDO1
IDO2
KIR3DL1
LAG3
LAIR1
LGALS9
NRP1
PD1LG2
PDCD1
TIGIT
TMIGD2
TNFRSF14
TNFRSF18
TNFRSF25
TNFRSF4
TNFRSF8
TNFRSF9
TNFSF14
TNFSF15
TNFSF18
TNFSF4
TNFSF9
VTCN1
